# Silicon Alters Leaf Surface Morphology and Suppresses Insect Herbivory in a Model Grass Species

**DOI:** 10.3390/plants9050643

**Published:** 2020-05-19

**Authors:** Casey R. Hall, Vaibhav Dagg, Jamie M. Waterman, Scott N. Johnson

**Affiliations:** Hawkesbury Institute for the Environment, Western Sydney University, Locked Bag 1797, Penrith NSW 2751, Australia; 18331211@student.westernsydney.edu.au (V.D.); j.waterman@westernsydney.edu.au (J.M.W.); scott.johnson@westernsydney.edu.au (S.N.J.)

**Keywords:** insect herbivores, silica, trichomes, epidermal cells, Poaceae, plant defence, Orthoptera, Lepidoptera

## Abstract

Grasses accumulate large amounts of silicon (Si) which is deposited in trichomes, specialised silica cells and cell walls. This may increase leaf toughness and reduce cell rupture, palatability and digestion. Few studies have measured leaf mechanical traits in response to Si, thus the effect of Si on herbivores can be difficult to disentangle from Si-induced changes in leaf surface morphology. We assessed the effects of Si on *Brachypodium distachyon* mechanical traits (specific leaf area (SLA), thickness, leaf dry matter content (LDMC), relative electrolyte leakage (REL)) and leaf surface morphology (macrohairs, prickle, silica and epidermal cells) and determined the effects of Si on the growth of two generalist insect herbivores (*Helicoverpa armigera* and *Acheta domesticus*). Si had no effect on leaf mechanical traits; however, Si changed leaf surface morphology: silica and prickle cells were on average 127% and 36% larger in Si supplemented plants, respectively. Prickle cell density was significantly reduced by Si, while macrohair density remained unchanged. Caterpillars were more negatively affected by Si compared to crickets, possibly due to the latter having a thicker and thus more protective gut lining. Our data show that Si acts as a direct defence against leaf-chewing insects by changing the morphology of specialised defence structures without altering leaf mechanical traits.

## 1. Introduction

Many grass species have the ability to hyperaccumulate silicon, which can make up to 10% dry weight in some species [1,2]. Silicon uptake has been shown to benefit plants under various stress conditions, including drought, salinity, heavy metals [3,4], pathogens [5] and herbivory [6,7]. Plants acquire Si from the soil in the form of silicic acid (Si(OH)_4_) which is then distributed throughout the plant [1]. Grasses can deposit Si as amorphous solid silica in various ways: inside cell walls and intercellular spaces, in silicified external structures such as trichomes and in specialised silica cells [8]. These Si-induced changes in leaf surface morphology are particularly effective against insect herbivores, reducing leaf consumption and hindering digestion [9,10]. Silica cell density in the leaf epidermis generally increases with Si availability, leading to tougher and more abrasive leaves and decreased herbivore performance [11,12]. Si can also be deposited in other surface structures such as trichomes, increasing their length and density [11,13]. Trichomes and leaf hairs are often sharp with rigid spikes, and have been shown to significantly damage herbivore digestive tracts [14]. When reinforced with Si, these surface structures cause even greater damage to the insect gut epithelium when ingested [15].

Si incorporated into plant tissues is assumed to increase mechanical defence traits by increasing leaf toughness and mechanical resistance. The accumulation of Si in the apoplast (in between the plasma membrane and cell wall) has been found to stabilise cell walls against damage [16,17,18]. Several studies have shown decreased electrolyte leakage (as a measure of membrane damage) in Si-supplemented plants under water stress conditions [19,20,21]. Si has also been found to reduce cell rupture of plant material ingested by insect herbivores, however, this was measured indirectly using chlorophyll released from damaged cells [9]. The Si-mediated reduction in cell membrane damage could reduce insect digestive efficiency through reduced cell rupture during digestion [9]. In contrast, other commonly used measures of leaf toughness, such as specific leaf area (SLA) and force to fracture, have shown no response to Si supplementation [22,23]. Very few studies have measured leaf mechanical traits in relation to Si supplementation and the effect on subsequent herbivore performance. As such, the specific mechanisms by which Si deters herbivory are not always clear, as the effect of Si on herbivores can be difficult to disentangle from Si-induced changes in leaf mechanical traits.

This study explores the effect of Si on leaf defence traits and herbivore growth using *Brachypodium distachyon* as a model grass. *Brachypodium distachyon* is used extensively as a model for pasture grass and cereal species [24,25]. In addition, *B. distachyon* has the ability to accumulate Si in similar amounts to other crop grasses [26], making *Brachypodium* useful for investigations of the role of Si in crops. *Brachypodium distachyon* develops two main types of nonglandular trichomes: macrohairs and prickle cells [13]. Recent work has shown that *B. distachyon* concentrates silicon deposition in the leaf macrohairs [13], and that Si supplementation in this species has significant negative effects on insect herbivory [27]. We tested the effect of Si in *B. distachyon* on two generalist insect herbivores: *Helicoverpa armigera* and *Acheta domesticus*. The cotton bollworm, *Helicoverpa armigera* (Lepidoptera, Noctuidae), is a highly polyphagous (over 180 host plants) major pest of crops worldwide [28], while the house cricket, *Acheta domesticus* (Orthoptera: Gryllidae), is omnivorous and also widespread. The two insects differ in their ability to extract nutrients and in the protection of the digestive tract. Lepidoptera have high gut pH that helps break down plant cell walls to extract nutrients, while Orthoptera have neutral gut pH and need to chew their food to break open cell walls [29]. The Orthoptera, however, have tougher periotrophic membranes (protective layer of the insect digestive tract), while lepidopteran gut linings are thinner and more easily damaged [30].

Our specific aims were to assess the effect of silicon on a) general mechanical leaf traits (specific leaf area, electrolyte leakage, dry matter content); b) the morphology of surface cells (macrohairs, prickle cells, Si cells and epidermal cells); and c) growth of two generalist herbivores. We hypothesised that: i) Si would reduce cell electrolyte leakage and increase leaf toughness and ii) increase the density and size of macrohairs, prickle cells and Si cells; and that iii) this would have a more negative effect on the growth of *H. armigera* compared to *A. domesticus* due to differences in digestion and gut anatomy.

## 2. Results

### 2.1. Effect of Si on Leaf Mechanical Traits

**Figure 1:**
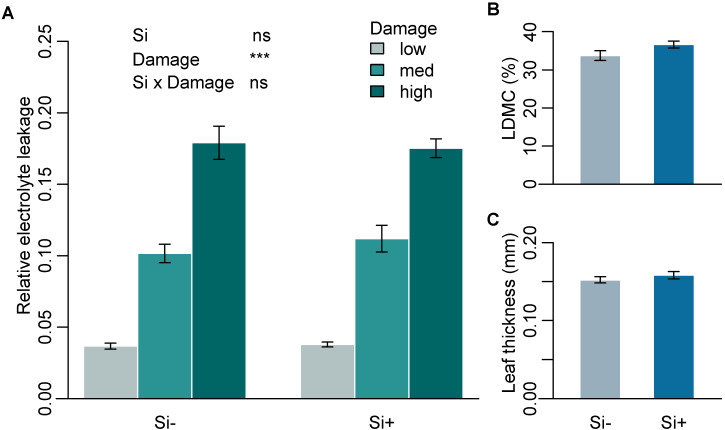
Mechanical leaf traits in response to Si supplementation. (**A**) Relative electrolyte leakage (REL) at three different levels of mechanical damage (*n* = 9), (**B**) percent leaf dry matter content (LDMC) (*n* = 12) and (**C**) leaf thickness (mm) (*n* = 9) of *B. distachyon* in Si+ and Si- plants. Values are means ± SE. Degrees of significance are indicated as follows: ns = not significant, *** *p* < 0.001.

Mean Si concentration in Si+ plants was 1.54% (± 0.14) while Si- plants were below the limit of quantification (<0.3%). To test the effect of Si on leaf toughness, we assessed three leaf mechanical traits that are commonly used as measures of leaf structure and toughness and are associated with defence against herbivory. Si had no significant effect on mechanical traits measured on leaves of *B. distachyon* (Table 1a). Higher levels of damage significantly increased relative electrolyte leakage (REL), supporting the use of REL as a reliable measure of cell rupture following mechanical damage. Si supplementation, however, had no significant effect on REL across all three damage treatments (Figure 1A and Supplementary Table S1). Si also had no significant effect on specific leaf area (SLA) or leaf thickness (Figure 1C and Table 1a). Si increased leaf dry matter content (LDMC), however, this was not significant (*p* = 0.055) (Figure 1B and Table 1a).

### 2.2. Effect of Si on Leaf Surface Morphology

**Figure 2:**
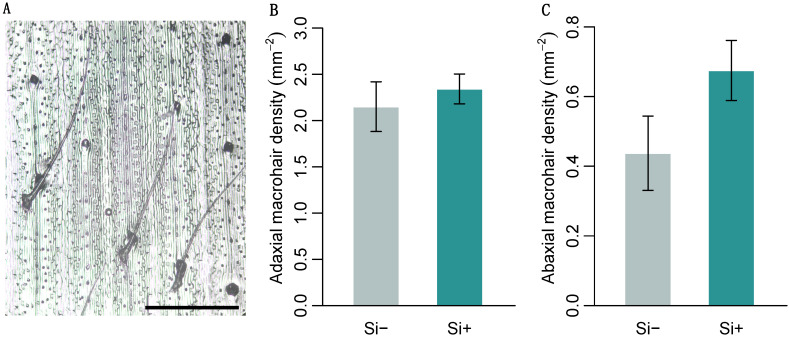
*Brachypodium distachyon* macrohair density in response to Si supplementation. (**A**) Image of macrohairs from epidermal leaf peels, scale bar: 0.5 mm. Macrohair density was quantified on (**B**) the adaxial (*n* = 12) and (**C**) abaxial leaf surfaces (*n* = 8–12). Values are means ± SE.

**Figure 3:**
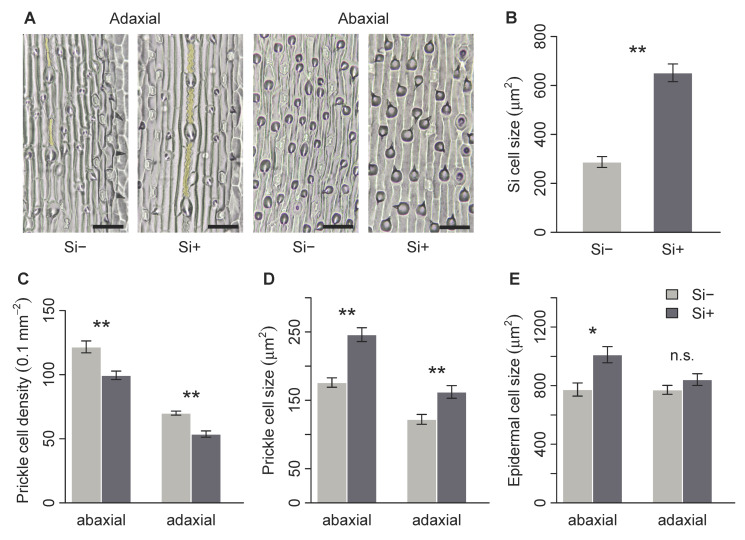
*Brachypodium distachyon* leaf surface morphology in response to Si supplementation. (**A**) images of adaxial and abaxial leaf surfaces; silica cells are false-coloured yellow, prickle cells are shown in dark grey, scale bars: 50 μm. (**B**) Silica cell size, (**C**) prickle cell density, (**D**) prickle cell size and (**E**) epidermal cell size on Si+ and Si- plants (*n* = 6). Values are means ± SE. Degrees of significance are indicated as follows: ns = not significant, * *p* < 0.05, ** *p* < 0.01.

*Brachypodium distachyon* contained macrohairs, prickle cells and silica bodies, the latter on the adaxial surface only (Figure 2A and Figure 3A). Macrohair density was higher on the adaxial surface, however, was not affected by Si (Figure 2B). Si increased abaxial macrohair density, however, this was not significant at *p* = 0.05 (Figure 2C and Table 1b). Only the adaxial surface contained visible Si cells, which were 127% larger in Si supplemented plants (Figure 3B and Table 1b). Both prickle cell density and size were higher on the abaxial surface (Figure 3 and Table 1b). Si increased the size of prickle cells by 33% and 40% on the adaxial and abaxial surfaces, respectively (Figure 3D and Table 1b). Prickle cell density, however, was significantly reduced by Si on both the adaxial and abaxial leaf surfaces (Figure 3C and Table 1b). We measured epidermal cell size to test if these changes were specific to specialised cells or more general changes in epidermal cell morphology. There was no difference in adaxial epidermal cell size, however, Si increased abaxial epidermal cell size by 31% (Figure 3E and Table 1b).

### 2.3. Effect of Si on Herbivore Performance

**Figure 4:**
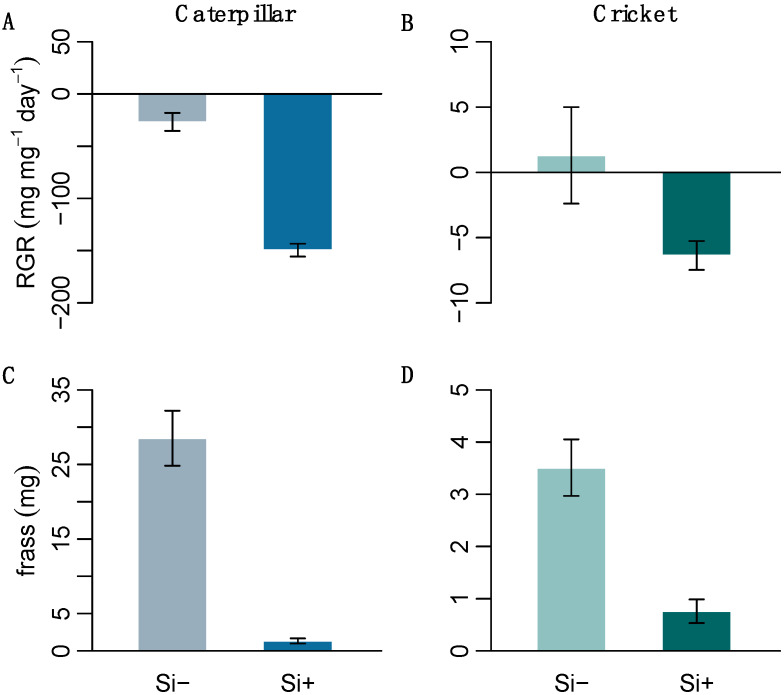
The relative growth rate of (**A**) *H. armigera* and (**B**) *A. domesticus* and their frass (**C**,**D**), respectively, after feeding on Si+ and Si- *B. distachyon* (*n* = 12). Values are means ± SE.

We performed herbivore feeding assays on two generalist insects with differing digestive abilities to test the effect of Si-induced changes in leaf morphology on insect herbivory. The resulting herbivore response was significantly negative; Si treatment decreased both caterpillar and cricket relative growth rates (RGR) (Figure 4 and Table 1c). Both caterpillars and crickets also produced significantly less frass on Si+ leaves (Figure 4 and Table 1c), suggesting that Si was acting as an effective feeding deterrent.

**Figure 5:**
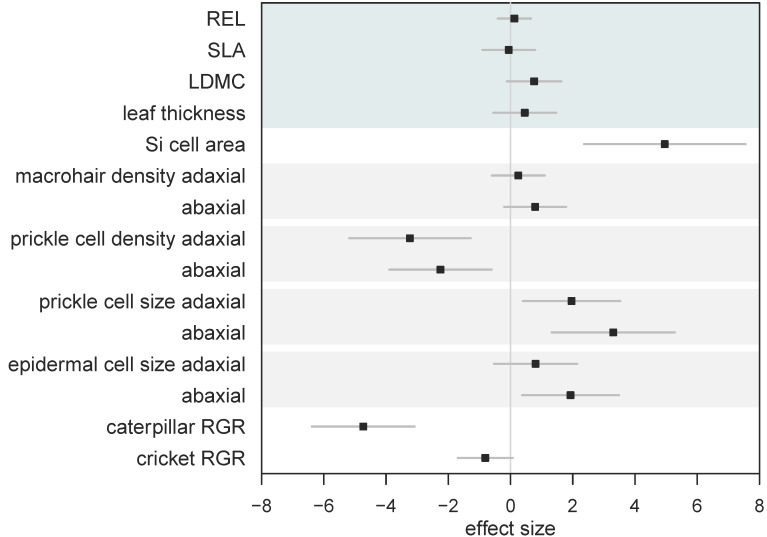
Standardised effect sizes (± 95% CI) of the influence of Si on leaf traits and herbivore growth. Whole leaf mechanical traits are grouped (top shaded area) for clarity. Shaded bars show pairings of adaxial and abaxial measurements. Positive effect size indicates higher values under Si supplementation. Bars that overlap zero indicate a nonsignificant effect.

We calculated standardised effect sizes to allow for the comparison of different traits within the same analysis (Figure 5). By comparing effect sizes, we found that overall Si had no effect on leaf mechanical traits, yet had significant and variable effects on leaf morphology, resulting in significantly reduced herbivore growth (Figure 5). However, the reduction in caterpillar RGR was significantly worse compared to cricket RGR when fed Si-supplemented plants (Figure 5).

## 3. Discussion

In this study, we show that silicon significantly alters the morphology of specialised surface defence structures in *B. distachyon*, resulting in reduced herbivore growth. However, the more commonly measured leaf mechanical traits associated with herbivore defence remained unchanged with Si addition. In *B. distachyon*, Si significantly increased the size of specialised silica cells and prickle cells. The significantly larger prickle and silica cells may explain the reduced palatability of Si-treated plants. Caterpillar growth was more negatively affected by Si compared to cricket growth (Figure 5), possibly due to differences in their digestive tracts, as crickets have a tough layer lining the digestive tract, protecting the gut lining from any abrasion caused by larger, more silicified prickle and silica cells. Our data shows that Si acts as a direct defence by changing the morphology of specialised defence structures without altering commonly measured antiherbivore leaf mechanical traits.

Contrary to our predictions, Si had no effect on measures of leaf toughness (SLA, LDMC and thickness) or cell leakage. SLA is often used as a measure of leaf toughness and is often correlated with defence against herbivory [31,32]. Previous research has also found no effect of Si on SLA measured across several grass species [23], while specific leaf mass (the inverse of SLA) also shows no response to Si in tall fescue [33]. We found that Si increased LDMC, however, this was marginally nonsignificant. LDMC is a good indicator of leaf structural tissue and palatability [34,35]. The inverse of LDMC is leaf water content which has been shown to both increase [23,36] and decrease [37] in response to Si supplementation. 

Our evidence suggests that Si has relatively minor effects on leaf mechanical traits in *B. distachyon* and is thus unlikely to explain dramatic negative effects of Si on herbivores. In this study, we used only indirect measures of leaf toughness (SLA, DMC and leaf thickness), possibly explaining the lack of any observed effect. However, a recent study found no effect of Si on overall leaf toughness when measured directly using biomechanical properties such as force to fracture [22]. Surprisingly, we found no effect of Si on cell rupture, measured through electrolyte leakage. This is in contrast to previous studies, where Si-supplemented plants had decreased electrolyte leakage [19,20,21] and decreased cell rupture, measured indirectly through chlorophyll content [9]. Plant species differ in not only their ability to accumulate Si but also where it is deposited [11]. Si can be deposited directly onto the cell wall matrix [38], where it protects the cell from damage and reduces cell rupture. In *B. distachyon*, however, the majority of Si deposition seems to occur in the macrohairs, although Si deposition in the epidermis was not visualised [13]. These interspecific differences in Si deposition may explain why we found no effect of Si on cell rupture. Additionally, the type of stress in this study (mechanical damage as opposed to salinity or pathogen infection) may obscure subtle differences in REL caused by membrane damage. 

The morphology and density of prickle and silica cells on the leaf epidermis of *B. distachyon* were altered in response to Si treatment. As we hypothesised, silica cells more than doubled in size in response to Si treatment, supporting previous studies that show increased epidermal Si deposition and larger silica cells in response to Si availability [11,18,39]. Silica cells are specialised epidermal cells that are actively filled with Si during leaf development [38]. Similarly, the size of prickle cells also increased in response to Si treatment. Previous work has found that these structures are filled with Si when available [11,38], potentially increasing cell size in the same way as silica cells. However, we also found that abaxial epidermal cells were larger on Si+ plants, thus it is unknown if the increased prickle cell size in this study is due to Si deposition in the prickle cells or generally larger epidermal cells. Various epidermal cell types have been shown to accumulate Si in the cell wall [8,40,41], potentially thickening the cell wall, which may explain increased epidermal cell size in Si+ plants. However, as mentioned above, epidermal Si deposition in *B. distachyon* has not been visualised. 

The growth of both herbivores was significantly reduced when fed Si-treated plants, however, caterpillars were more negatively affected. Previous studies have shown that Si has a negative effect on *H. armigera* growth [6,27]. Orthopterans also respond negatively to Si [12], but are not affected to the same extent as lepidopterans [23]. The significantly reduced frass collected from both insects on Si+ plants suggests that Si is acting as a feeding deterrent. Even reduced amounts of ingested Si+ plant matter could still affect digestion. Ultimately, impaired digestion could lead to reductions in development and reproduction, however, longer feeding trials are needed to assess the effect of Si on insect life history traits. Orthoptera generally have a less permeable gut membrane (peritrophic membrane) compared to lepidoptera [30], which in theory should provide greater protection from mechanical damage from silicified cells and trichomes. Trichomes play an important role in plant defence, and have been shown to significantly damage the peritrophic matrix of caterpillars [14]. A recent study found that silicified trichomes caused significant damage to the mid- and hind-gut wall in caterpillars and that experimental removal of these trichomes improved caterpillar growth [15]. It is likely that the larger silica cells, prickle cells and silicified macrohairs in Si+ *B. distachyon* caused significant damage to the peritrophic matrix of *H. armigera*. It remains to be seen to what extent silicified cells or trichomes damage orthopteran gut linings. 

## 4. Conclusions

Our data show that Si acts as a direct defence against herbivory by changing the morphology of specialised defence structures. These changes alter the leaf surface, reducing leaf palatability and the growth of insect herbivores. In contrast, we found that Si had no effect on leaf mechanical traits commonly associated with plant defence; these traits are thus unlikely to contribute to the dramatic negative effects of Si on herbivory. The different responses of crickets and caterpillars in this study suggest that digestive anatomy plays a role in insect susceptibility to plant Si and highlight the lack of knowledge on the effect of silicified defence structures on insect gut membranes.

## 5. Materials and Methods

### 5.1. Plant Growth Conditions

Plant cultivation and experiments were conducted in a single glasshouse chamber maintained at 22/18 °C day/night on a 14:10 h cycle with natural lighting throughout the experiment. Humidity was controlled at 50% (± 6%).

Seeds of *Brachypodium distachyon* (Bd21-3) were obtained from the French National Institute for Agricultural Research (INRA, Versailles, France). Seeds were grown hydroponically following a previously published protocol [27]. Briefly, seeds were soaked in water for 2 h to soften the lamella and palea which were then removed using forceps. Seeds were then sterilised in a solution of 0.9% sodium hypochlorite and 0.1% Triton X-100 for 30 min and washed several times with water before being inserted into perlite irrigated with half-strength nutrient solution (as described below). After stratification at 4 °C for 7 days, plants were grown for 10 days to achieve uniform seedling growth and transplanted to hydroponics with three seedlings per cup. The hydroponics setup consisted of two nested disposable cups that contained a foam disc cut to size with three slots cut to hold plants (see Figure S1). Each cup was filled with approximately 330 mL of full strength nutrient solution, as per Jung et al. [42] except Fe(III) EDTA concentration was doubled due to iron deficiencies detected in trial plants.

### 5.2. Experimental Design

Silicon treatments (Si+) used liquid potassium silicate (K_2_SiO_3_) (Agsil32, PQ Australia, Adelaide, Australia) at a concentration of 2 mM (SiO_2_ equivalent) added to the nutrient solution. Controls (Si-) had potassium chloride (KCl) added to balance additional K+ and Cl- in the Si+ treatments. The pH of both solutions was adjusted to pH 5.5 using HCl. Solutions were replaced weekly and cups were rotated. Plants were grown hydroponically for a further 6 weeks prior to the start of the experiment. A total of 24 cups containing two plants each were randomly assigned to either silicon or control resulting in 12 replicates (cups) per treatment. 

### 5.3. Leaf Mechanical Traits

We measured three leaf traits that are relevant to herbivory. Specific leaf area (SLA), leaf thickness and dry matter content (LDMC) can be considered measures of leaf structure and toughness and are associated with defence against herbivory [34,43]. All leaf trait measurements were performed on the second fully expanded leaves from three tillers from one plant per cup. Leaf thickness was measured using digital callipers (± 0.02 mm). Three measurements per leaf were made, avoiding the mid rib, and the average value used in subsequent analysis. SLA was calculated using leaf area measured in ImageJ (version 2.0.0) [44] on scanned freshly harvested leaves. Sampled leaves were dried at 40 °C for 24 h and weighed. SLA was calculated as leaf surface area (mm^2^) divided by leaf dry weight (mg). Percent LDMC was measured by weighing freshly sampled leaves, which were then oven-dried at 40 °C for 24 h and reweighed. LDMC was calculated as the difference between fresh and dry weight then divided by fresh weight and converted to percentage. 

Relative electrolyte leakage (REL) was measured in nine plants from each Si treatment using methods modified from Feng et al. [45]. In brief, six fully developed leaves were removed from one plant per cup. Using dressing forceps, in sets of two, leaves were damaged one of three ways: 1) neither leaf was damaged (low), 2) one leaf was pinched 20 times (medium), or 3) both leaves were pinched 20 times (high). Each set of leaves was submerged in 10 mL H_2_O for 1 h at 25 °C and electrical conductivity (EC1) was recorded using a ST300C conductivity meter (Ohaus, USA). Leaves were transferred to a 100 °C water bath for 5 min and then cooled until 25 °C. Electrical conductivity was remeasured (EC2). REL was calculated as EC1/EC2.

### 5.4. Leaf Surface Morphology

An additional 24 replicates (12 in each treatment) were prepared using the same methods as above for leaf surface morphology measurements. The second fully expanded leaf from one plant per cup was sampled and frozen for macrohair density counts. Abaxial and adaxial surfaces of each leaf were photographed under 10× magnification. The cell counter function in ImageJ (version 2.0.0) [44] was used to count macrohairs in a 3 × 5 mm area in the middle of each leaf. The second fully expanded leaf from the second plant in each cup was removed and kept in a ziplock bag with moist cotton to maintain leaf moisture for leaf peels. Leaf epidermal peels were made of the adaxial and abaxial side of the middle of each leaf within two hours of samples being taken. Clear varnish was painted onto the centre of each leaf, allowed to dry, removed with clear tape and mounted on a microscope slide. Prickle cells were counted (0.2 mm^2^ field of view) and average basal cell area calculated from five prickle cells per sample using ImageJ (version 2.0.0) [44]. A single row of silica cells, approximately 0.4 mm long, from each sample was measured for average cell area. The area of five epidermal cells was averaged per sample. Only cells fully contained within the image frame were included in the respective analysis.

### 5.5. Silicon

Harvested plants were dried for three days at 40 °C and ball milled for further analysis. Approximately 80 mg of ground leaf material was analysed to measure Si concentration using X-ray fluorescence spectrometry (Epsilon 3x; PANalytical, EA Almelo, The Netherlands). Si standards were prepared by mixing SiO_2_ with methyl cellulose in the range of 0–10% Si. Measurements were calibrated using certified plant material (NCS ZC73018 Citrus leaves, China National Institute for Iron and Steel).

### 5.6. Insect Performance

Relative growth rates of *Helicoverpa armigera* larvae and *Acheta domesticus* nymphs were determined based on Slansky [46]. *Helicoverpa armigera* caterpillars (supplied by CSIRO Agriculture & Food, Narrabri, Australia) were reared on artificial diet until 4th instar. *Acheta domesticus* nymphs (4th instar) were obtained from a commercial supplier (BioSupplies, Yagoona, Australia) and kept in ventilated plastic containers containing dry grass mixture and cardboard. All insects were starved for 24 h and weighed before being placed in a container with a tiller of fresh leaf material with the cut end inserted into a 1.5 mL tube with water to maintain freshness. Insects were kept at 22 °C and allowed to feed for 48 h, after which time they were starved for a further 24 h to allow the frass to pass, before being reweighed. All frass was collected, dried and weighed. Relative growth rate (RGR), defined as body mass growth relative to initial body mass, was calculated as: mass gained (mg)/initial mass (mg)/time (days).

### 5.7. Statistical Analyses

All analyses were performed in R (version 3.5.0) [47]. The effect of Si supplementation on all leaf trait measures (except REL) and herbivore growth measures were assessed using Mann–Whitney–Wilcoxon tests due to non-normality of data. REL was analysed using a generalised linear model with Si and damage as fixed effects and a Gamma distribution due to heavily skewed data and heteroscedasticity. The model was checked for overdispersion and significance values generated using likelihood ratio test (chi-squared approximation). In order to compare the magnitude of the effect of Si supplementation on plant traits and herbivore growth, we calculated the standardised effect sizes (SES) for all traits as standardised mean difference (SMD) = ((µ1 − µ2)/s) (µ1 = mean trait value of Si+ plants, µ2 = mean trait value of Si- plants, s = standard deviation) using the *effsize* package in R [48]. Standardised effect sizes allow for the comparison of different traits within the same analysis. A 95% confidence interval bar that does not overlap zero indicates a significant effect of Si supplementation; positive effect sizes indicate a positive effect of Si on the measured trait. 

## Figures and Tables

**Table 1:** Mann–Whitney–Wilcoxon results of the effect of Si on leaf traits and herbivore responses. Values are means ± standard error (*n* = 6–12).

	Si-	Si+	*W*	*p*
**(a) Leaf Mechanical Traits**				
Si (% DW)	ND (<0.3)	1.54 ± 0.14	-	-
Mean relative electrolyte leakage	0.10 ± 0.01	0.11 ± 0.01	-	-
Specific leaf area (mm^2^ mg^−1^)	3.6 ± 0.1	3.6 ± 0.2	85	0.47
Leaf thickness (mm)	0.15 ± 0.004	0.16 ± 0.004	28	0.29
Dry matter content (%)	33.7 ± 1.28	36.6 ± 0.92	38.5	0.055
**(b) Surface Morphology**				
Macrohair density (mm^2^) adaxial	2.15 ± 0.27	2.34 ± 0.16	48	0.17
abaxial	0.44 ± 0.12	0.68 ± 0.09	24	0.068
Si cell area (μm^2^)	287.2 ± 22.2	651.6 ± 36.2	0	0.002 *
Prickle cell density (0.1 mm^−2^) adaxial	70.0 ± 1.6	53.7 ± 2.5	36	0.005 *
abaxial	121.7 ± 4.6	99.5 ± 3.3	35	0.004 *
Prickle cell size (μm^2^) adaxial	122.1 ± 7.2	162.2 ± 9.3	2	0.009 *
abaxial	176.0 ± 6.9	246 ± 10.1	0	0.002 *
Prickle cell coverage (%) adaxial	8.6 ± 0.6	8.6 ± 0.3	16	0.82
abaxial	21.4 ± 1.1	24.5 ± 1.4	7	0.093
Epidermal cell size (μm^2^) adaxial	771.3 ± 30.7	841.5 ± 39.8	10	0.240
abaxial	773.6 ± 44.9	1011.3 ± 55.2	3	0.015 *
**(c) Herbivore Response**				
*H. armigera* RGR (mg mg^−1^)	−26.8 ± 8.7	−149.5 ± 6.1	144	<0.001 *
*A. domesticus* RGR (mg mg^−1^)	1.3 ± 3.7	−6.4 ± 1.1	125	0.002 *
*H. armigera* frass (mg)	28.5 ± 3.7	1.3 ± 0.4	144	<0.001 *
*A. domesticus* frass (mg)	3.5 ± 0.5	0.8 ± 0.2	129	0.001 *

Significant terms are indicated by an asterisk (* *p* < 0.05).

## Supplementary Materials

The following are available online at https://www.mdpi.com/2223-7747/9/5/643/s1, Figure S1: Hydroponics system and dimensions, Table S1: Effects of Si and damage level on *B. distachyon* relative electrolyte leakage.
