# Supplementary material for: Silicon Alters Leaf Surface Morphology and Suppresses Insect Herbivory in a Model Grass Species
Source: Plants (Basel). 2020 May 19;9(5):643. doi: 10.3390/plants9050643 (PMC7285219; doi:10.3390/plants9050643)
Supplement: Supplementary file 1 [file plants-09-00643-s001.pdf]

Table S1. Effects of Si and damage level on *B. distachyon* relative electrolyte leakage. Generalized linear models P-values were calculated based on likelihood ratio tests using chi-squared test statistic. Significant values ($P < 0.05$) are highlighted in bold.

Relative Electrolyte Leakage	d.f.	χ^2	*p*
Si	1,48	0.02	0.88
Damage	2,48	159.14	< **0.001**
Si x damage	2,48	0.41	0.52

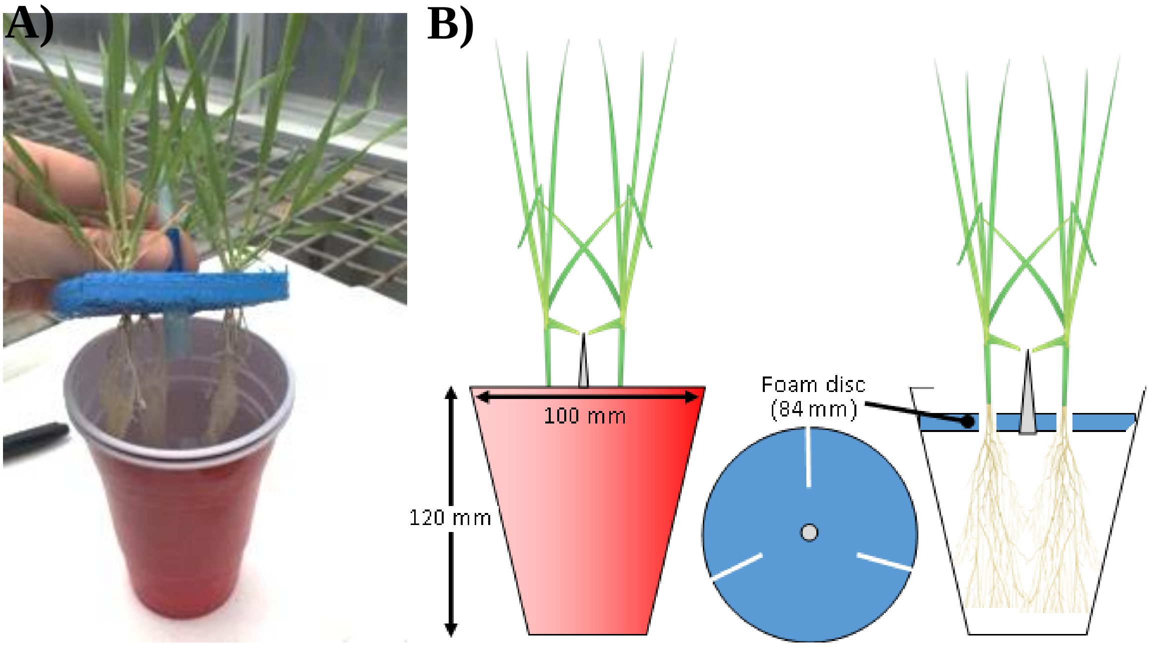

Figure S1. (**A**) a photograph showing the hydroponics system used and (**B**) the dimensions.
